# Advantages and robustness of partial VMAT with prone position for neoadjuvant rectal cancer evaluated by CBCT-based offline adaptive radiotherapy

**DOI:** 10.1186/s13014-023-02285-6

**Published:** 2023-06-17

**Authors:** Zhe Zhang, Shou Yu, Feng Peng, Zhibo Tan, Lei Zhang, Daming Li, Pengfei Yang, Zhaoming Peng, Xin Li, Chunfeng Fang, Yuenan Wang, Yajie Liu

**Affiliations:** 1 Department of Radiation Oncology, Peking University Shenzhen Hospital, Shenzhen, China; 2 Shenzhen-Peking University, Hong Kong University of Science and Technology Medical Center, Shenzhen, China; 3 Department of Radiation Oncology, Hebei Yizhou Cancer Hospital, Zhuozhou, China

**Keywords:** Offline ART, Prone position, Neoadjuvant rectal cancer, Partial VMAT

## Abstract

**Background and purpose:**

This study aims to explore the advantages and robustness of the partial arc combined with prone position planning technique for radiotherapy in rectal cancer patients. Adaptive radiotherapy is recalculated and accumulated on the synthesis CT (sCT) obtained by deformable image registration between planning CT and cone beam CT (CBCT). Full and partial volume modulation arc therapy (VMAT) with the prone position on gastrointestinal and urogenital toxicity, based on the probability of normal tissue complications (NTCP) model in rectal cancer patients were evaluated.

**Materials and methods:**

Thirty-one patients were studied retrospectively. The contours of different structures were outlined in 155 CBCT images. First, full VMAT (F-VMAT) and partial VMAT (P-VMAT) planning techniques were designed and calculated using the same optimization constraints for each individual patient. The Acuros XB (AXB) algorithm was used in order to generate more realistic dose distributions and DVH, considering the air cavities. Second, the Velocity 4.0 software was used to fuse the planning CT and CBCT to obtain the sCT. Then, the AXB algorithm was used in the Eclipse 15.6 software to conduct re-calculation based on the sCT to obtain the corresponding dose. Furthermore, the NTCP model was used to analyze its radiobiological side effects on the bladder and the bowel bag.

**Results:**

With a CTV coverage of 98%, when compared with F-VMAT, P-VMAT with the prone position technique can effectively reduce the mean dose of the bladder and the bowel bag. The NTCP model showed that the P-VMAT combined with the prone planning technique resulted in a significantly lower complication probability of the bladder (1.88 ± 2.08 vs 1.62 ± 1.41, *P* = 0.041) and the bowel bag (1.28 ± 1.70 vs 0.95 ± 1.52, *P* < 0.001) than the F-VMAT. In terms of robustness, P-VMAT was more robust than F-VMAT, considering that less dose and NTCP variation was observed in the CTV, bladder and bowel bag.

**Conclusion:**

This study analyzed the advantages and robustness of the P-VMAT in the prone position from three aspects, based on the sCT fused by CBCT. Whether it is in regards to dosimetry, radiobiological effects or robustness, P-VMAT in the prone position has shown comparative advantages.

**Supplementary Information:**

The online version contains supplementary material available at 10.1186/s13014-023-02285-6.

## Background

External radiation therapy prior to surgery is the standard treatment for non-metastatic locally advanced rectal cancer [1]. For these patients, a dose of 25 × 2 Gy is usually given clinically to reduce the stage and improve the complete resection rate [2]. However, some dose-related toxicity to the organs at risk (OARs) is inevitable during pelvic radiotherapy, especially for the bladder and the small intestine [3]. At present, some methods are available to reduce the dose toxicity of OARs, such as the use of intensity-modulated radiotherapy (IMRT) or volume-modulated arc therapy (VMAT) optimization, and the use of the prone position to reduce the irradiated volume of OARs [4–6]. However, to make full use of these techniques, it is necessary to locate more accurately the target area and the anatomy of the OARs at each treatment. Image guidance of cone-beam computed tomography (CBCT) provides great significance for the validation of the location repeatability and the analysis of the volume changes of the surrounding organs in patients with locally advanced rectal cancer [7]. Some studies have shown that the actual irradiation dose received by patients is different from the planned dose, due to target dislocation caused by changes in bladder and rectal volume [8]. CBCT is used to reduce target site misalignment in these moving organs. However, great changes in OARs volumes were observed during the treatment, with a variation in bladder coefficient of up to 58% per CBCT [9], and a variation in bowel bag volume of 28.5% [5]. At present, many studies have applied CBCT to the adaptive radiotherapy process, and deformed image registration (DIR) of planned CT and CBCT to form sCT is a commonly used method [10, 11]. DIR is a process of registering an image data-set to a reference image set, which can be used for recalculation and dose accumulation. Adaptive radiotherapy can be more individualized and customized for dose optimization and program selection, and can also retrospectively analyze the difference between the actual irradiation dose received by the patients and the planned dose [12]. At last, the dose volume histogram (DVH) was used to calculate the probability of normal tissue complications (NTCP) [13–15]. In this study, we used full VMAT (F-VMAT) and partial VMAT (P-VMAT) techniques, in the prone position, to design treatment planning for patients with rectal cancer. Then, sCT generated from CBCT and planning CT were used to conduct re-calculation for offline adaptive radiotherapy. Ultimately, this study was to investigate the incidence of gastrointestinal (GI) and urogenital (GU) toxicity in patients with rectal cancer in the prone position, after recalculation based on the NTCP model.

## Methods

This study retrospectively analyzed 31 patients with rectal cancer treated with the VMAT technology in our hospital from 2020 to 2022. All patients underwent computed tomography (CT) in the prone position and were scheduled for radiotherapy. In the prone position, a Klarity Prone Pelvis System (Klarity Medical&Equipment Co.Ltd., GZ, China) was applied to allow the abdomen to extend into its aperture. Each patient was asked to drink 600 cc of water before measuring bladder volume. These values were then recorded prior to simulated positioning. The BVI 9400 ultrasound scan was used for all bladder measurements by placing the probe approximately 3 cm above the epiphysis and applying ultrasound gel. The probe should be oriented towards the bladder and there is also a visual simplified graphical aid to verify the bladder. Patients underwent weekly CBCT scans and were asked to drink the same volume of water at approximately the same time prior to treatment, within ± 100 cc of the bladder volume measurement and the measurement at positioning, before treatment could proceed. Patients were also advised to avoid high-fiber foods and gas-producing diets during the course of treatment, in order to avoid intestinal gas filling the bowel bag, and thus changing the volume of the target organ [4]. Patients with rectal gas excess after CBCT scan were instructed to undergo either protective care or rectal gas deflation, followed by image-guided radiation therapy (IGRT).

**Fig. 1:**
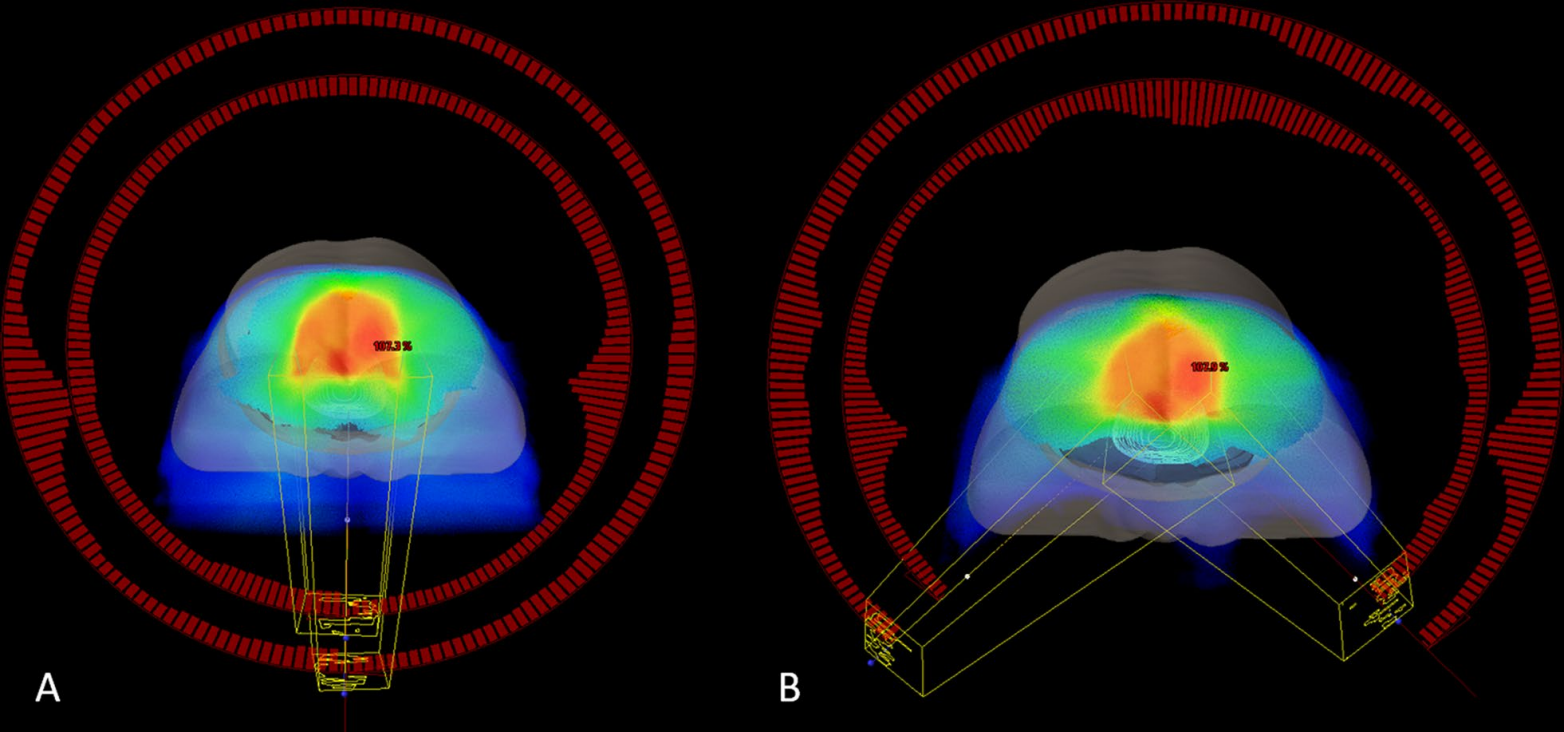
Treatment planning design for two techniques: **A** F-VMAT; **B** P-VMAT

GTV was defined as tumor and positive lymph nodes. The reference scan revealed the tumor itself, but no increased dose was applied to the tumor. For CTV, the mesorecta, presacral space, internal iliac lymph node region, and, if applicable, the obturator lymph node region, were delineated by one radiation oncologist [16]. According to the geometric uncertainty reported by Nijkamp et al. [6], the mesentery is divided into upper and lower parts, with the transition at the base of the bladder, enabling the distinction of the edge between the upper and lower mesentery. Target volumes, such as clinical target volume (CTV) and total tumor volume (GTV), OARs (i.e., the bladder, bowel bag for small bowel and femur heads) were contoured by qualified radiation oncologists according to RTOG guidelines [17]. The planned target volume (PTV) and planned total tumor volume (PGTV) were generated by adding a 5 mm edge to the CTV and GTV, respectively. The prescriptions were 50 Gy and 45 Gy in 25 fractions to the PGTV and PTV respectively. The full arc VMAT plans were designed as two full arcs from 179° to 181° and reversed, and the partial arc VMAT plans were designed as two arcs from 135° to 225° and reversed, as shown in Fig. 1. The plans used the method of Jaw tracking, and the initial width of X direction was no more than 15 cm. The collimators were all set as 355° and 5° respectively. The prescribed dose should cover 95% of PTV and/or PGTV. When making the plans, the same optimization constraints were set for two different plans for each patient. The optimization constraints were somewhat different for these 31 patients, but we made plans that were clinically appropriate after performing a quantitative analysis of clinical normal tissue impact (QUANTEC) analysis [18, 19]. The optimization constraints are shown in Additional file 1.

**Fig. 2:**
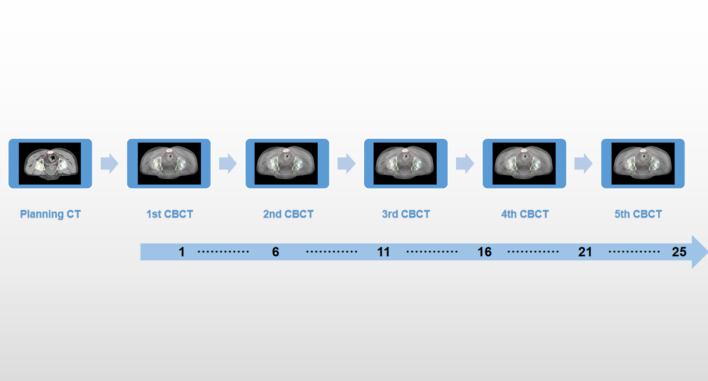
Imaging study protocol (computed tomography [CT] simulation, and cone beam computed tomography [CBCT]) that was used during the treatment course at Peking University Shenzhen Hospital

**Fig. 3:**
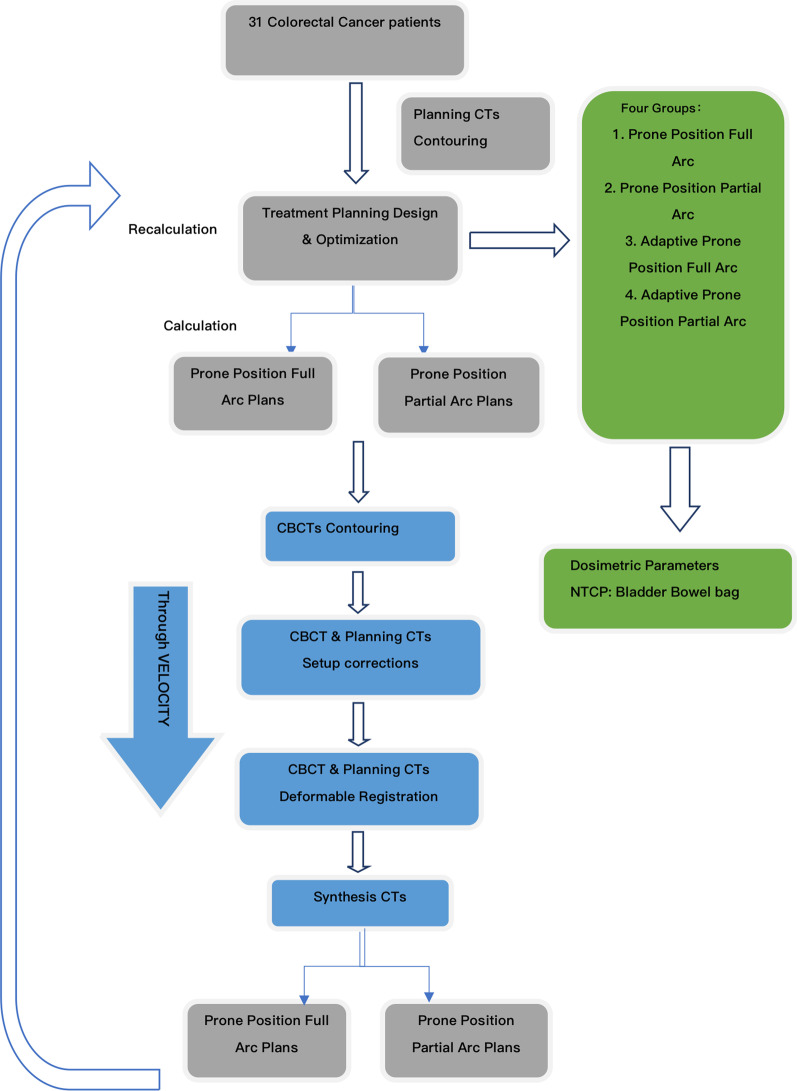
Workflow of the planning design and optimization, image deformable registration, dose accumulation for comparison

Patients enrolled in this study underwent CBCT scan acquisition once a week, as shown in Fig. 2. OARs were contoured for each CBCT. To avoid inter observer variation, the target volume and target volume of planned CT and CBCT were delineated by the same radiation oncologist. It is worth noting that the images in this study were corrected for electron density, and the electron density of each organ was obtained via the AXB 13.5 version. The dose calculation was also performed using the AXB 13.5 algorithm. Images were transferred from the Eclipse 15.6 treatment planning system to the Velocity software V4.0 (Varian Medical Systems, Palo Alto, CA) for image registration and dose reconstruction. The accuracy of deformable image registration and dose reconstruction using computer simulations and patient data has been demonstrated in previous papers, and has been tested and validated separately [20, 21]. The clinical shift was used for rigid alignment between partial KVCBCT images and the corresponding planned CT. Using Velocity’s “CBCT corrected multi-pass deformation”, “CBCT corrected single-pass deformation” and “deformable multi-pass” algorithms, the planned CT was deformed into KVCBCT, and 155 sCT images were generated by combining actual anatomy for dose calculation. CT images were planned to be repaired with anatomical structures in the field of view of CBCT with a custom length of 27.6 cm × 27.6 cm, and manual cross-check was performed when necessary. The treatment dose was recalculated on these sCT images to represent the actual patient dose, including setup corrections and anatomical changes. CBCT was performed only once a week, and on days when no CBCT scans were performed, the fractional dose was calculated by default by selecting the last acquired CBCT fused with sCT, assuming that organ deformation remained similar. By aggregating all fractional doses, dose accumulation was achieved for PGTV, PTV to organs at risk such as bowel bag, bladder, femoral head and bone marrow. The entire workflow is shown in Fig. 3.

The dose parameters were read out through the clinical protocol template on Eclipse 15.6. The main dosimetry indicators included the coverage of CTV, the mean dose of each OARs, and the dose of each evaluation index. Radiobiological indicators included the bladder and bowel bag NTCP, to quantify the risk of late injury of OARs. The NTCPs were calculated using the linear-quadratic Poisson model and the Lyman-Kutcher Burman model. DVH was imported into the Matlab-based internal programs (MathWorks, Natick, MA) to calculate the NTCP. The radiobiology parameters and endpoints used for NTCP calculations were specified in previous studies [14, 22], and Parameter estimates of the LKB model fitted to the late GU toxicity data (grade > 1 toxicity within two years) as a = 2, TD50 = 80 Gy, γ50 = 4.

$$EUD = \left( {\sum V_{i} \cdot D_{i}^{a} } \right)^{\frac{1}{a}}$$

here a is a unitless model parameter that is specific to the normal structure or tumor of interest, V_i_ is unitless and represents the ith partial volume receiving dose D_i_ in Gy.

$$NTCP = \frac{1}{{1 + \left( {\frac{{TD_{50} }}{EUD}} \right)^{{4{\upgamma }50}} }}$$

where TD50 is the dose leading to a 50% chance of controlling the tumor, TD50 is the tolerance dose for a 50% complication rate of the normal structure at a specific time interval, γ50 is a specific parameter that describes the slope of the dose–response curve. The following parameter values were used.

Considering the NTCP of Bowel bag, the final endpoint evaluated was RTOG grade 2 acute GI toxicity (comprising diarrhea, abdominal pain and mucous discharge). The most significant volumetric factor was fit to a normal tissue complication probability (NTCP) curve in the form of a logistic-type function. The model from Roeske et al. was:

$$NTCP = \frac{1}{{1 + (V_{50} /V)^{k} }}$$

where V_50_ = 410 cm^3^, V is the volume receiving 45 Gy and k = 3.2 [14].

With two treatment plans and ten offline adaptive plans per patient, we grouped patients using the partial arc plan with those using the full arc plan. The difference between the partial arc plans before and after the adaptive plan dose overlay was used as the absolute difference ΔD for the plan robustness assessment. Similarly, the plan robustness of the full arc technique plan was obtained. The main indicators were Dmean in the target area and organs at risk and V15, V30, V45 in the bowel bag and NTCP in the bladder and bowel bag [18].

**Fig. 4:**
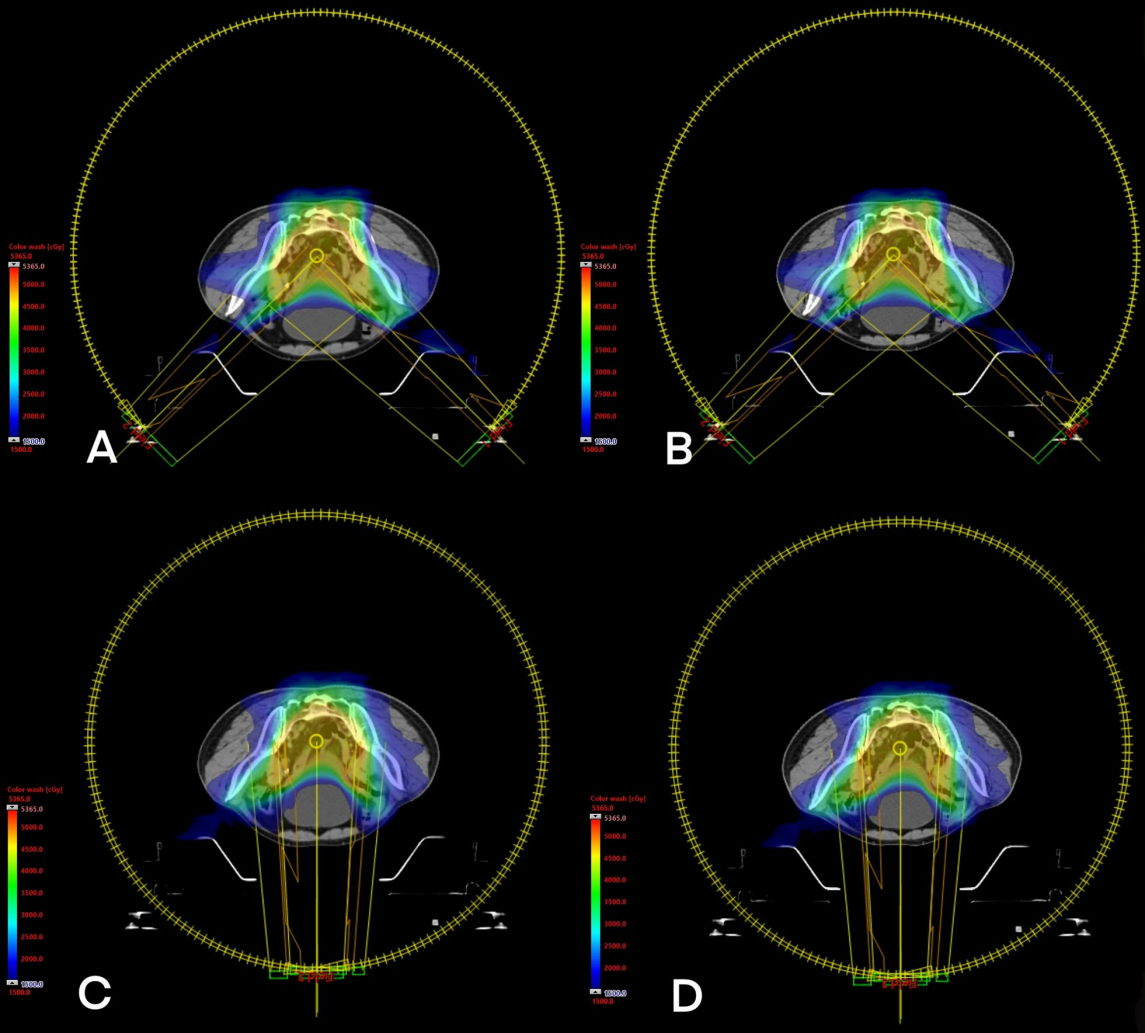
The dose distribution of treatment planning design for two techniques (**A**, **B** P-VMAT; **C**, **D** F-VMAT) and adaptive dose (**A**, **C**) and un-adaptive dose (**B**, **D**) respectively

Comparison of planned and cumulative doses to target volume and organs at risk. In the case of normally distributed parameters, paired t-test was used for the confirmation of statistical significance. For non-normally distributed parameters, Wilcoxon matched-pairs signed-rank test (*) was used instead. A two-sided *P* < 0.05 was considered statistically significant. Declared values were mean ± standard deviation (Fig. 4).

## Result

### Dose evaluation

After the accumulated adaptive dose based on CBCT, the CTV coverage rate of F-VMAT was still the highest (98.89%), and P-VMAT reached 98.75%. The difference between P-VMAT and F-VMAT was significant (*P* ≤ 0.001). However, the coverage of CTV reached over 95%, which could meet the clinical requirement. After dose accumulation, the mean bladder dose of P-VMAT was 23.92 Gy, which was the lowest dose in the two planning methods, with no significant difference (*P* = 0.054). The mean dose of P-VMAT in the bowel bag was also the lowest at 8.88 Gy, and there was a significant difference between P-VMAT and F-VMAT (*P* ≤ 0.001). The V30 of the bowel bag also differs significantly in P-VMAT (161.42 cc ± 112.46) and F-VMAT (189.2 cc ± 127.56) planning techniques. The mean dose of bone marrow P-VMAT planning method had no significant difference compared with F-VMAT (*P* = 0.538), and at the left and right femoral heads, it was the average dose obtained by F-VMAT that was more advantageous in both cases, with significant differences observed in both as well (Table 1).

**Table 1:** Summary of the dosimetric parameters, radiobiological indices for Full and Partial techniques after accumulated adaptive plans

		Full	Partial	ρ_(Wilcoxon)_ < 0.05
PTV	Dmean (Gy)	48.16 ± 1.06	47.73 ± 1.07	0.000
GTV	Dmean (Gy)	52.28 ± 0.64	51.85 ± 0.53	0.001
CTV	Dmean (Gy)	48.74 ± 1.09	48.36 ± 1.07	0.000
	Coverage	98.89 ± 1.99	98.75 ± 2.12	0.000
Bladder	Dmean (Gy)	24.44 ± 4.40	23.92 ± 4.28	0.054
	gEUD	9.81 ± 4.75	9.22 ± 3.93	0.021
	NTCP	1.88 ± 2.08	1.62 ± 1.41	0.041
Bowel bag	Dmean (Gy)	9.63 ± 6.85	8.88 ± 6.16	0.000
	V45	66.60 ± 52.71	62.84 ± 47.89	0.000
	V30	189.2 ± 127.56	161.42 ± 112.46	0.000
	V15	503.53 ± 314.70	423.28 ± 257.14	0.000
	NTCP	1.28 ± 1.70	0.95 ± 1.52	0.000
Bone Marrow	Dmean (Gy)	24.69 ± 1.45	24.72 ± 1.54	0.538
Femoral-Head-L	Dmean (Gy)	14.60 ± 2.07	15.03 ± 1.83	0.000
Femoral-Head-R	Dmean (Gy)	14.92 ± 2.01	15.20 ± 2.01	0.026

### Biological effects

**Fig. 5:**
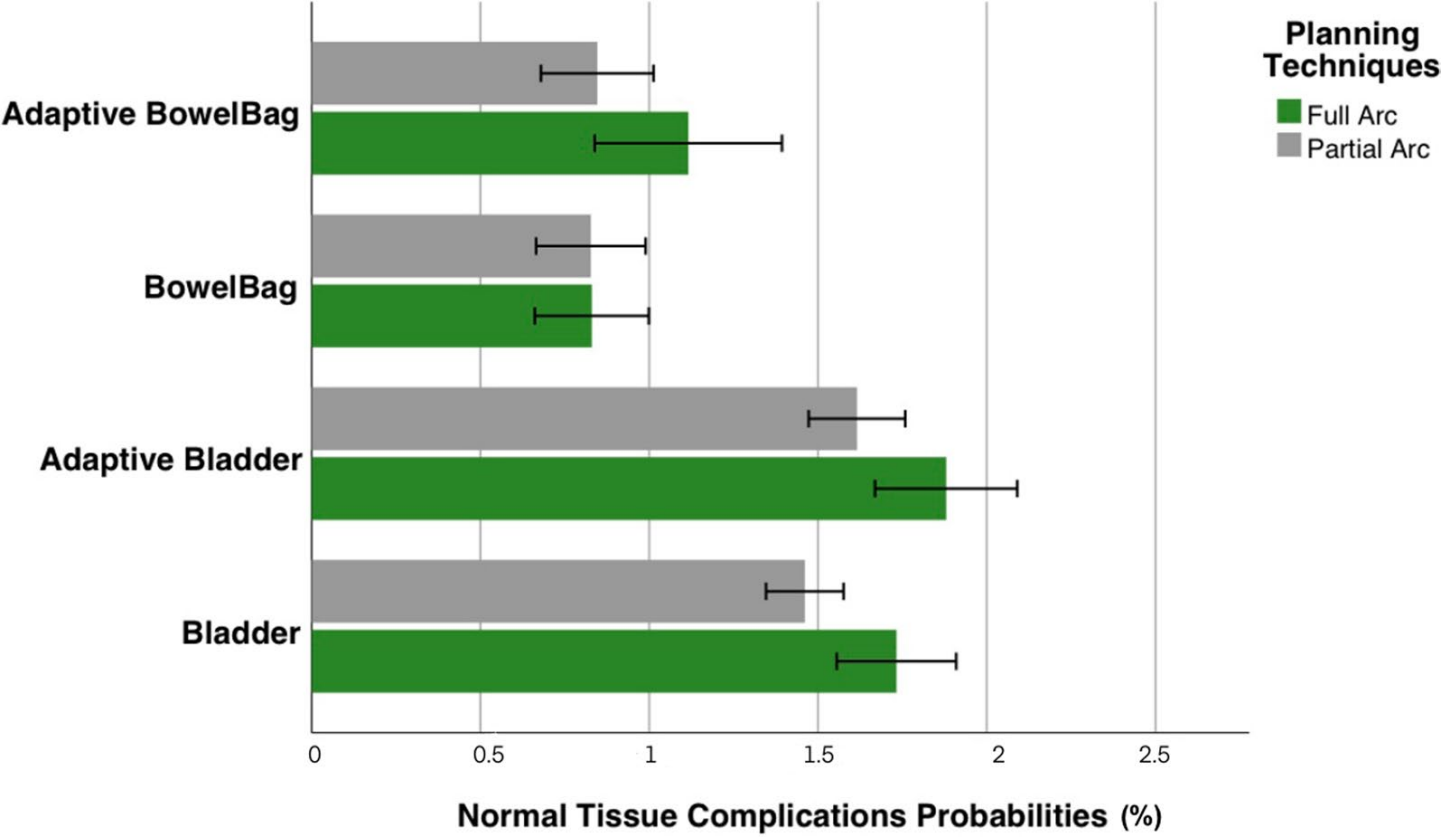
The normal tissue complication probabilities of bladder and bowel bag by using full arc and partial arc, with or without adaptive radiotherapy

For biological effects, this study analyzed the bioequivalent dose of two vital organs. The bioequivalent dose of the bladder was fitted to the late GU toxicity data based on the LKB model (> grade 1 toxicity within two years). The NTCP of bladder with F-VMAT (1.88 ± 2.08) was higher than that with P-VMAT (1.62 ± 1.41), and there was statistical difference (*P* = 0.041). Considering the NTCP of the bowel bag, the final endpoint was RTOG grade 2 acute gastrointestinal toxicity (including diarrhea, abdominal pain and mucus excretion). The F-VMAT (1.28 ± 1.70) plan was significantly higher than P-VMAT (0.95 ± 1.52), with a P value of less than 0.001 (Fig. 5).

### Plan robustness

We also evaluated the robustness of the radiotherapy plan. For these two plans, it was obvious that the robustness obtained by the P-VMAT planning method was stronger, and the dose modification of each organ at risk was smaller, with statistical differences, except for the Dmean of the bowel bag and the left and right femoral heads (Table 2). Dose indexes with main differences included Dmean of ΔCTV, coverage of ΔCTV, V30, V45 of Δbowel bag, Dmean of Δbowel bag, and Dmean of Δbone marrow. Dmean of ΔCTV is 0.14 ± 0.76 in the P-VMAT and 0.47 ± 0.74 in the F-VMAT (*P* < 0.001). The V30 of the Δbowel bag was also highly variable, 12.66 ± 34.95 in F-VMAT versus 3.62 ± 6.29 in P-VMAT. Bladder’s ΔDmean is 0.06 ± 2.47 in the P-VMAT and 0.36 ± 2.45 in the F-VMAT (*P* = 0.007). The same can be obtained in terms of equivalent dose. NTCP of the Δbladder increased by 0.15 ± 0.61 in the P-VMAT and 0.15 ± 0.56 in the F-VMAT, with a statistical difference (*P* = 0.009). The changes observed in the Δbowel bag were more obvious. P-VMAT and F-VMAT increased by 0.08 ± 0.14and 0.12 ± 0.16 respectively (*P* < 0.001).

**Table 2:** The summary of dose difference between treatment plans and adaptive plans for dosimetric parameters, radiobiological indices

		Full	Partial	ρ_(Wilcoxon)_ < 0.05
ΔPTV	Dmean (Gy)	0.36 ± 0.83	0.002 ± 0.854	0.000
ΔGTV	Dmean (Gy)	0.25 ± 0.57	0.001 ± 0.47	0.008
ΔCTV	Dmean (Gy)	0.47 ± 0.74	0.14 ± 0.76	0.000
	Coverage	− 1.00 ± 1.93	− 1.12 ± 2.07	0.001
ΔBladder	Dmean (Gy)	0.36 ± 2.45	0.06 ± 2.47	0.007
	gEUD	0.29 ± 1.47	0.32 ± 1.71	0.003
	NTCP	0.15 ± 0.56	0.15 ± 0.61	0.009
ΔBowel bag	Dmean (Gy)	0.35 ± 0.66	0.30 ± 0.76	0.096
	V45	4.72 ± 11.25	1.04 ± 5.65	0.000
	V30	12.66 ± 34.95	3.62 ± 6.29	0.000
	V15	13.15 ± 83.08	6.62 ± 16.58	0.164
	NTCP	0.12 ± 0.16	0.08 ± 0.14	0.000
ΔBone Marrow	Dmean (Gy)	0.62 ± 0.51	0.53 ± 0.55	0.000
ΔFemoral-Head-L	Dmean (Gy)	0.47 ± 0.83	0.51 ± 0.96	0.795
ΔFemoral-Head-R	Dmean (Gy)	0.56 ± 0.70	0.64 ± 0.88	0.786

## Discussion

First of all, the dose obtained by the offline adaptive radiotherapy based on CBCT was more accurate. While it is not as accurate as the daily online adaptive radiotherapy generated using CBCT for each treatment plan, it still allows for retrospective and closer analysis of the doses received in practice. The use of DIR recalculation in each CBCT of patients undergoing neoadjuvant radiotherapy for rectal cancer allowed the present study to circumvent part of the effects for setup error correction and anatomical alterations. In addition, this approach played an important role in obtaining the realism of dosimetric parameters and led to an NTCP-based analysis of possible clinical toxicity in patients. Finally, based on robustness, we analyzed the effect of full-arc and particle-arc planning on target area and OAR in prone colorectal cancer patients.

In recent years, more and more off-line adaptive methods have been used for dosimetric and robustness evaluation. In 2021, Buranaporn et al. [4] studied the relationship between dose-received volume and complications of OARs during radiotherapy for bladder cancer, and the applied method was the fusion of CBCT and PCT using DIR for off-line adaptive radiotherapy. Tamihardja et al. [23] used CBCT-based off-line adaptive radiotherapy to assess the difference between actual dose and planned dose in prostate cancer. Wang et al. [24] study also compared dosimetry differences between daily and weekly CBCT using an MVCBCT-based off-line adaptive approach. Regarding algorithms, the AXB 13.5 calculates more accurately the air cavity than the traditional AAA algorithm calculation [25]. Because the gas contained in the rectum and other organs of the patients can vary greatly, therefore the AXB 13.5 was much closer to the actual dose distribution.

In this study, two different planning methods were used. In prone rectal cancer, the P-VMAT technique showed more obvious advantages in the protection of major OARs, including the bladder and bowel bag. Holyoake et al. [26] conducted a meta-analysis to investigate the mean difference in intestine volume at different dose levels between grade 0–2 toxicity and grade 3 toxicity, as well as the toxicity risk of V10Gy and V40Gy receiving normally graded radiotherapy. In all the included studies, patients also received chemotherapy simultaneously. They found evidence of significant dose-volume-toxic effects of a large number of clinically relevant doses in the treatment of rectal cancer. In contrast to our results, different delineations of the bowel bag (intestinal loop and pouch) caused inconvenience. Abraham’ et al. [18] study was shown that there was a significant dose-volume relationship between small bowel irradiation and late small bowel toxicity in rectal cancer patients who underwent surgery after neoadjuvant radiotherapy. Limiting V30 to < 200 cm^3^ reduces the incidence of grade 3 or higher late small bowel toxicity to < 5% (after surgery). In this study, we added constraints for bowel bag at the planning design stage, with a mean V30 of 161.42 cm^3^ with P-VMAT and 189.2 cm^3^ with F-VMAT, and mean V30 of majority of patients were restricted to less than 200 cm^3^. For advanced bladder toxicity, the significance of dose reduction is not as clear. Fiorini et al. [27] concluded that only high doses (> 60–65 Gy) to small volumes, and 50–60 Gy to the full bladder, increased the risk of moderate to high toxicity, and different treatment sites were analyzed. These dose-volume-toxic effects exceed clinically relevant doses for rectal cancer. The differences noted between planned dose volume and actual dose volume may be the result of fractional changes in rectal and bladder volumes observed in our study. This is consistent with the findings of Oscar et al. [9] who reported substantial intercellular changes in the bladder. Nassef et al. [28] also showed that, although patients were instructed to keep their bladder full during intensity modulated radiotherapy treatment (IMRT), the relative change in bladder volume was as high as 39%. Therefore, in our study, the addition of P-VMAT to prone colorectal cancer patients was proposed, and a more realistic off-line adaptive radiotherapy dose assessment was performed. The results showed that P-VMAT showed good effects not only for the dose of OARs, but also for the bioequivalent doses. P-VMAT was also superior to F-VMAT, and the doses obtained in the OARs and target areas were closer to the real dose irradiation. For both methods, the Dmean and NTCP of the bladder and the bowel bag were statistically different. To obtain a more accurate planning study, the robustness and complexity of the plan needs to be analyzed quantitatively. In 2020, Hernandez et al. [29] suggested that the distribution was not exactly the dose delivered to the patient due to uncertainties in dose calculation and treatment delivery, including variations in patient’s setup and anatomy. With robustness assessment, one can evaluate the change in dose distribution compared to the nominal dose by recalculating the schedule obtained under different errors. Robustness optimization and evaluation is typically set to several problems; for example, allows for setting errors, range uncertainty, respiratory control, and possible anatomical variations (e.g., cavity filling and tumor shrinkage) to reduce the need for replanning. There are many studies on planning robustness evaluation, but there are few studies on offline adaptive robustness evaluation combined with comparison of planning methods. In a study, Tamihardja et al. [23] conducted evaluation of VMAT robustness in prostate cancer and off-line adaptive evaluation of CBCT. Zhang et al. [10] study also showed that rectal NTCPs in prostate cancer are highly affected by dose-to-dose variability in FCBCT-based soft tissue matching. In this study, the results were the same. NTCP variability in the bladder and bowel bag had a great impact, especially in the bowel bag, where NTCP was reduced to a certain extent.

Several limitations are present in this study. Firstly, instead of daily CBCT scan and dose accumulation of each fraction, it was performed once a week. Such a way of doing cannot truly reflect the status of treatment of that time, which is an inaccurate adaptive radiotherapy dose reconstruction. Also, the quality of the CBCT influences the robustness of the dose accumulation. In the process of image acquisition, the maximum 18 cm wide field of view of CBCT is inevitably smaller than the field of view planned by CT. We were only able to perform a partial image fusion, but the high-dose area covered all the target areas and OARs within the field of view of CBCT in our study. Due to the retrospective design of our study, inter-observer differences in CT and CBCT cannot be avoided, especially regarding the upper and lower boundaries of organ resection. However, we still insisted on assigning only one radiation oncologist to conduct the delineation of the target area, so that the analysis can be more repeatable. The image contouring and DIR between CBCT and CT also had certain errors. Although a density assignment as correction strategy was applied, Dunlop et al. [30] showed an absolute mean dose error of 0.7% for pelvic CBCT-based step-wise dose calculation in case of an anterior–posterior distance < 25 cm. In this study, the robustness of two separate plans were proposed. The offline adaptive method was used to verify the plan's robustness. Above all, the comparison of the dose of the main OARs can better help us to choose techniques that are more appropriate, execution and dosing-wise, to the planned dosing. In future studies, the two techniques should be compared based on clinical data from complication follow-up. To explore whether the robustness of clinical data based on the probability of complications of this approach is also stronger than that of full-arc irradiation, the results could provide a more adequate clinical theoretical basis for clinical planning of the prone partial-arc technique.

## Conclusion

In summary, the method of P-VMAT irradiation with the prone position was proposed in this study, and its advantages and robustness based on CBCT image fusion of OARs were analyzed. More specifically, the advantages of the prone position of P-VMAT in terms of dosimetry, radiobiological effects and robustness were analyzed. Radiobiological analysis showed that the NTCP of the bowel bag and bladder obtained by partial arc irradiation was lower based on sCT obtained by CBCT& Planning CT fusion.

## Supplementary Information

**Additional file1**. **Table 1**. Plan optimization objectives for target and organs at riskusing dose–volume parameters. Dmax is maximum point dose; Dvolume%, dose received by % of the structure volume; VD, percentage of organ volume receiving D Gy; Dp is prescribed dose.

## Data Availability

All datasets provided in this study are included in the article or Additional file 1.
