# Supplementary material for: Advantages and robustness of partial VMAT with prone position for neoadjuvant rectal cancer evaluated by CBCT-based offline adaptive radiotherapy
Source: Radiat Oncol. 2023 Jun 17;18:102. doi: 10.1186/s13014-023-02285-6 (PMC10276517; doi:10.1186/s13014-023-02285-6)
Supplement: Supplementary file 1 — Additional file1. Table 1. Plan optimization objectives for target and organs at riskusing dose–volume parameters. Dmax is maximum point dose; Dvolume%, dose received by % of the structure volume; VD, percentage of organ volume receiving D Gy; Dp is prescribed dose. [file 13014_2023_2285_MOESM1_ESM.docx]

| PTV/OAR |  | Dose–volume constraints |
| --- | --- | --- |
| PTV |  | D100%>45Gy  Dmax<(Dp x 1.05)Gy |
| PGTV |  | D100%>50Gy  Dmax<(Dp x 1.05)Gy |
| Bladder |  | D50%<25Gy  Dmean<25Gy |
| Bowel Bag |  | Dmax<49.5Gy  V15<50%  V30<20% |
|  |  | V45<10% |
| Bone Marrow |  | Dmean<25Gy  V30<50% |
| Femoral-Head-L |  | Dmean<15Gy  Dmax<49Gy |
| Femoral-Head-R |  | Dmean<15Gy  Dmax<49Gy |

Table 1. Plan optimization objectives for target and organs at risk (OAR) using dose–volume parameters. Dmax is maximum point dose; Dvolume%, dose received by % of the structure volume; VD, percentage of organ volume receiving D Gy; Dp is prescribed dose.
